# Confirmed local endemicity and putative high transmission of *Schistosoma mansoni *in the Sesse Islands, Lake Victoria, Uganda

**DOI:** 10.1186/1756-3305-4-29

**Published:** 2011-03-01

**Authors:** Claire J Standley, Moses Adriko, Fred Besigye, Narcis B Kabatereine, Russell J Stothard

**Affiliations:** 1 Wolfson Wellcome Biomedical Laboratory, Department of Zoology, Natural History Museum, London, SW7 5BD, UK; 2 School of Biology, University of Nottingham, Nottingham, NG7 2RD, UK; 3 Vector Control Division, Ministry of Health, P.O. Box 1661, Kampala, Uganda; 4 Center for Neglected Tropical Disease Control, Liverpool School of Tropical Medicine, Liverpool, L3 5QA, UK

## Abstract

The Sesse Islands, in the Ugandan portion of Lake Victoria, have long been considered a low transmission zone for intestinal schistosomiasis. Based on observations of high prevalence of *Schistosoma mansoni *infection in the northern-most islands of this archipelago, a follow-up survey was conducted to ascertain whether transmission was endemic to this island group, combining parasitological and malacological surveys. Prevalence of intestinal schistosomiasis was again observed to be high, as was intensity of infections which, combined with low reported incidence of treatment, suggests that chemotherapy-based control initiatives are not being maximally effective in this region as high levels of population movement between islands and districts are confounding. The local disease transmission was confirmed by the observations of high abundance of *Biomphalaria*, as well as field-caught snails shedding *S. mansoni *cercariae. DNA sequencing of 12 cercariae revealed common mitochondrial *cox*1 haplotypes, as well as, novel ones, consistent with the high genetic diversity of this parasite in Lake Victoria. Intestinal schistosomiasis is firmly endemic in parts of the Sesse Islands and more broadly, this island group provides an insight into the future challenges to be faced by the Ugandan National Control Programme in regularly reaching these rather remote, inaccessible and largely itinerant communities.

## Findings

The Sesse Islands, in the Ugandan portion of Lake Victoria, have long been considered a low transmission zone for intestinal schistosomiasis, although until recently, few if any formal surveys had been undertaken in this archipelago. The disease is caused by the trematode parasite *Schistosoma mansoni *which, as part of its life cycle, requires a suitable freshwater snail intermediate host (of the genus *Biomphalaria*); the predominantly exposed, sandy beaches of the Sesse Islands, mostly almost devoid of vegetation, were thought to be unfavourable for such snails, thus limiting the potential transmission of the disease. In 2003, the Ugandan Ministry of Health initiated a National Control Programme, consisting of mass drug administration (MDA) with praziquantel (PZQ) to school-age children in high-prevalence and high-risk districts, based on baseline data from rapid mapping surveys [1,2]. Kalangala District, which is synonymous with the Sesse Islands, was not included in the first roll-out of MDA but was added in 2005 as more information became available upon local disease burdens [3].

As part of a larger effort to monitor the efficacy and coverage of the National Control Programme, in January 2010 the Global Network for Neglected Tropical Diseases (GNTTD: http://globalnetwork.org/) funded an ambitious study to attempt to survey all islands in the Ugandan part of Lake Victoria which revealed an extremely heterogeneous distribution of intestinal schistosomiasis in school-aged children on the Sesse archipelago [4]. Notably, prevalence of infection was particularly high in the northern-most islands of this group which form part of Bufumira sub-county. However, the January surveys in Bufumira did not include malacological sampling, and thus could not shed light on whether local transmission was occurring *in situ*. Moreover, the survey, as well as previous rapid mapping exercises of the Ugandan Lake Victoria shoreline, revealed high levels of community itinerancy, with a substantial percentage of children travelling frequently between islands and the mainland [5]. As such, it was therefore not possible to confirm whether intestinal schistosomiasis was indeed locally acquired and thus firmly endemic to Bufumira sub-county, or whether the observed high prevalence might be attributed to migrants importing cases from other island groups or the mainland.

To clarify the possibility of high transmission and local endemicity in this part of the Sesse Islands, in November 2010 a combined parasitology and malacology expedition was conducted throughout the northern islands of Bufumira sub-county, revisiting the sites that had been mapped in January earlier in the year. As with the previous survey, a lot quality assurance sampling method (LQAS) was attempted [2], whereby approximately 15 children were selected for rapid assessment of the prevalence of intestinal schistosomiasis at a particular locality to ascertain if disease prevalence exceeded a high prevalence threshold (i.e. ≥50%). Each child was asked to provide a single urine and a single stool sample: the urine sample was used to test for the presence of schistosome circulating cathodic antigen (CCA) using a rapid diagnostic lateral flow test (Rapid Medical Diagnostics, Pretoria, South Africa) while the stool sample was used to make double Kato-Katz thick smears. CCA tests were scored by eye as 'negative', 'trace', 'single positive', 'double positive' or 'triple positive', with all results bar 'negative' being considered infection positive. Kato-Katz thick smears were examined for counts of *S. mansoni *eggs. Egg counts were multiplied by 24 for estimation of numbers of eggs per gram of faeces (EPG). Infection intensity classes were based on WHO recommendations, whereby <100 EPG is considered a 'light' infection, 100-400 EPG is a 'moderate' infection and >400 EPG is a 'heavy' infection [6]. Each child was also asked to participate in a short questionnaire, which asked: 1) place of birth; 2) length of residence in Kalangala District; 3) history of previous treatment with PZQ and 4) knowledge of what is intestinal schistosomiasis. All children were given treatment with PZQ after participating in the survey, as well as, albendazole for soil-transmitted helminthiasis. Written informed consent was obtained from a parent, as well as the participating child, prior to inclusion in the survey.

**Figure 1:**
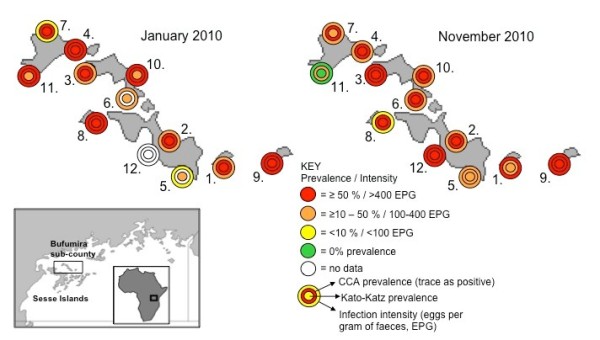
**Sketch map of the Sesse Islands (inset) in relation to Lake Victoria, with close up views of the findings of the mapping surveys undertaken in Bufumiri sub-county in January 2010 (left) and November 2010 (right)**. The islands within this sub-county lie in a NW-SE orientation covering a 25 km distance. With one or two exceptions, the prevalence of intestinal schistosomiasis is broadly high (>50%) across the within sampled children from the 12 shoreline communities.

The follow-up survey's findings confirmed the observation of high prevalence of intestinal schistosomiasis in this portion of Bufumira sub-county, based on 11 of the sites first surveyed in January 2010, plus an additional location (Table 1 and Figure 1). Indeed, in several cases prevalence was higher than in the previous survey; this was somewhat surprising, given that the previous survey was also intended to check up on delivery of praziquantel to the islands and administration of the drug to children in the local schools/settlements. In fact, only 29.5% of children reported having received praziquantel. Intensity of infection was also still high in the follow-up survey, and sometimes greater than previously, suggesting that the control programme was not being maximally effective in this region, potentially due to the logistical difficulties and financial costs associated with travelling between islands to deliver training and drugs. One exception was Luwungulu landing site (Map ID 11 in Figure 1) where both prevalence and intensity notably decreased between January and November.

**Table 1:** Prevalence and intensity of intestinal schistosomiasis across the northern Sesse Islands, based on Kato-Katz double thick smears and CCA urine lateral flow tests (trace test results were considered infection-positive) of the sampled children across 12 shoreline sites.

**Site (Map ID) *GPS coordinates***^***o***^	Kato-Katz double thick smears				CCA urine lateral flow test		
	Number positive	Number sampled	Prevalence (95% CI)	Mean EPG* (95% CI)	Number positive	Number sampled	Prevalence (95% CI)
Banda (1)			22.2	564.0			40.9
*S0.25887;E32.39511*	4	18	(6.4-47.6)	(76.0-1052.0)	9	22	(20.7-63.6)
Bosa (2)			66.6	216.0			55.0
*S0.18037;E32.29378*	12	18	(40.9-86.6)	(61.0-371.0)	11	20	(31.5-76.9)
Kaaya (3)			62.5	505.2			62.5
*S0.18955;E32.30222*	10	16	(35.4-84.8)	(116.7-893.7)	10	16	(35.4-84.8)
Kachanga (4)			84.2	267.0			83.3
*S0.18037;E32.29378*	16	19	(60.4-96.6)	(128.3-405.7)	15	18	(58.5-96.4)
Kafuna (5)			30.0	176.0			41.6
*S0.26450;E32.37188*	3	10	(6.6-65.2)	(0.0-417.5)	5	12	(15.1-72.3)
Kagonya (6)			78.95	199.2			66.6
*S0.21350;E32.33266*	15	19	(54.4-93.9)	(103.5-294.9)	14	21	(43.0-85.4)
Kammesse (7)			40.00	120.0			66.6
*S0.16811;E32.27144*	4	10	(12.1-73.7)	(28.4-211.6)	10	15	(38.3-88.1)
Kibibi (8)			50.0	96.0			66.6
*S0.22536;E32.30767*	1	2	(1.2-98.7)	(NA^$^)	4	6	(22.2-95.6)
Kitobo (9)			78.9	894.4			80.9
*S0.26241;E32.42812*	15	19	(54.4-93.9)	(400.2-1388.6)	17	21	(58.0-94.5)
Misonzi (10)			60.8	252.9			54.5
*S0.19313;E32.32238*	14	23	(38.5-80.2)	(107.6-398.1)	12	22	(32.2-75.6)
Luwungulu (11)			0.0	NA			20.0
*S0.19292;E32.26009*	0	10	(0.0-30.8)		2	10	(2.5-55.6)
Sserinya (12)			65.0	457.8			57.1
*S0.26071;E32.35516*	13	20	(40.7-84.6)	(182.2-733.5)	12	21	(34.0-78.1)

TOTAL			58.1	384.8			59.3
	107	184	(50.6-65.3)	(486.3-283.3)	121	204	(52.2-66.1)

* Calculated as the arithmetic mean of infected patients (i.e. EPG >0), ^$ ^Confidence intervals were not able to be calculated as there was only one infected individual at the site.

As with previous demographical surveys, a substantive level of population itinerancy was observed among the surveyed schoolchildren, with 56.3% reporting to have been born outside Kalangala District, and 49.5% having lived at that site for less than three years; 16.7% had been resident for less than a year, putting these interviewed children at risk of missing mass drug administration initiatives which are only carried out once each year.

In contrast to the January 2010 survey of Bufumira sub-county, the follow-up expedition also included a malacological element. At 15 sites, 12 of which were associated with a location also examined for prevalence of intestinal schistosomiasis in local school-aged children, the shoreline of Lake Victoria was surveyed for the presence and abundance of *Biomphalaria *snails, and other freshwater gastropods. Sampling effort was quasi-standardized by having the same collector scooping with a long-handled mesh sieve at each site, and quantified by limiting scooping time to 30 minutes per locality, across a 25-metre stretch of shoreline. Environmental variables, such as habitat type, depth, substrate and water chemistry values, were also recorded for each locality. If two or more habitats were observed at a site, they were surveyed separately and given a letter to designate the different habitats, such as with B005a and B005b. All *Biomphalaria *snails observed were collected, placed in transparent plastic cups filled with mineral water and exposed to sunlight, to check for infection with trematode larvae. Emerging schistosome cercariae were individually harvested and placed on Whatman FTA Indicator cards (Whatman plc, Maidstone, UK), their genomic DNA extracted using published protocols [7], and the ASMIT fragment of the mitochondrial *cox*1 gene amplified and sequenced [8]. The edited sequences were compared to an existing database of 138 *S. mansoni *haplotypes from Lake Victoria and Lake Albert [8-10].

**Figure 2:**
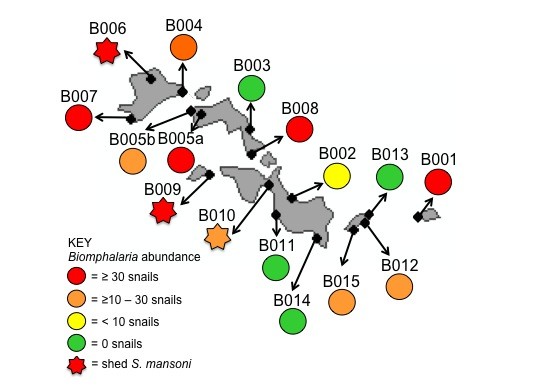
**Sketch map of the islands within Bufumiri sub-county and distribution and abundance of *Biomphalaria *at each of the 15 sampled sites. Note that at site B005, snails could be found in marshy fringes (B005a) as well as within the lake proper (B005b)**. Snails shedding schistosome cercariae were found at 3 sites (B006, B008 and B010) clearly affirming the local risk-of-infection with *S. mansoni *for people conducting water contact at these sites.

Contrary to previous surveys elsewhere in the Sesse Islands, large numbers of *Biomphalaria *were found, at several of the surveyed locations inclusive of numerous other freshwater snail species (Figure 2). The maximum number of *Biomphalaria *collected from a single location was 94, at site B007. Moreover, at three of the sites, *Biomphalaria *snails were found shedding *S. mansoni *cercariae clearly demonstrative of parasite transmission occurring *in situ*; prevalence of infected snails ranged from 1.3% at site B009 to 37.5% at site B010. 12 individual cercariae were successfully sequenced, and their haplotypes corresponded to two haplotypes commonly found throughout Lake Victoria (H1 and H23, Genbank accession numbers GQ415163 and GQ415192 respectively), as well as, two novel haplotypes, named H139 and H140 (Genbank accession numbers HQ839767 and HQ839768). This is consistent with sampling elsewhere in Lake Victoria, where the high genetic diversity of the parasite frequently results in new haplotypes being observed; the identification of H1 and H23 suggests that the Sesse Islands are part of the wider Lake Victoria 'population' of *S. mansoni*, as the parasite shows little population genetic structure throughout the region, potentially due to the mixing effects of human population movement and migration [10].

The relationship between abundance of *Biomphalaria *and prevalence of infected snails was tested statistically against the environmental observations also recorded using a multivariate linear regression model; increased abundance of *Biomphalaria *was positively associated with low turbulence/sheltered habitats, suggesting that the exposed beaches elsewhere in the Sesse Islands might be contributing to the low numbers of snails (and low prevalence of intestinal schistosomiasis) seen there. No other variables were significantly associated with abundance or incidence of infected snails.

Overall, the combination of parasitological and malacological surveys has clearly confirmed the endemicity of intestinal schistosomiasis to certain parts of the Sesse Islands, and emphasized the importance of maintaining control initiatives in Kalangala District. The high prevalence of the disease, combined with the observation of snails shedding various haplotypes of *S. mansoni*, suggest that Bufumira sub-county may be a local hotspot for intestinal schistosomiasis, and contradicts earlier evidence suggesting that this western portion of Lake Victoria is a much lower transmission environment than the eastern regions [5]. In fact, it could be said that these Sesse Islands epitomize the present and future obstacles faced by the National Control Programme throughout Uganda; the islands are only accessible by boat, and frequent fuel shortages, with continuously increasing prices, further challenge the best efforts of health officers to provide regular treatment to local schools and health centers. Moreover, this archipelago, like much of the rest of lacustrine Uganda, is characterized by highly motile and itinerant fishing communities, whose frequent movements not only assist in spreading intestinal schistosomiasis between different parts of the shoreline, but may also result in their children missing annual mass drug administration, time and time again. These island communities in Lake Victoria make up a substantial proportion of the population at risk of contracting intestinal schistosomiasis, yet these populations have been largely ignored in region-wide predictive mapping initiatives [11,12]. Since suitable snail habitats, and thus local transmission and exposure risk, are heterogeneously distributed, region-wide approaches need to be bolstered with smaller scale mapping efforts like this for better and more accurate identification of local treatment needs, particularly so in an island group like the Sesse.

## Competing interests

The authors declare that they have no competing interests.

## Authors' contributions

The study was conceived by CJS, NBK and JRS. Fieldwork was undertaken by CJS, MA, FB and JRS; CJS, MA and FB collected parasitological samples and MA and JRS carried out malacological surveys. Molecular and statistical analyses were done by CJS; the manuscript was prepared by CJS and JRS, and all authors read and approved the final version.
